# Supplementary material for: Cognitive and neural mechanisms of mental imagery supporting creative cognition
Source: Commun Biol. 2025 Sep 30;8:1386. doi: 10.1038/s42003-025-08513-x (PMC12484779; doi:10.1038/s42003-025-08513-x)
Supplement: Supplementary file 2 — Supplementary Information [file 42003_2025_8513_MOESM2_ESM.pdf]

Cognitive and neural mechanisms of mental imagery supporting creative cognition

Supplementary Information

Methods:

Quality control for semantic network construction

In Study 2, due to the generation of multiple texts by participants, the study opted to aggregate the 10 texts from each condition into a single longer text for the calculation of semantic features. When calculating the semantic network robustness, it was considered that a participant needs to construct two semantic networks, and the size of the network could be a latent variable affecting its robustness[1]. There may be a greater number of associations with more words, making the network less vulnerable to attack. To control for the influence of word count, it was necessary to standardize the weights of the semantic network: the ratio of word count produced by each participant under the two conditions was calculated (ratio = N_{MI}/N_{SU}). N represents word count. The network weights under MI condition (correlation matrix) were then standardized ($weighted\_matrix\_normalized_{MI}$ = $weighted\_matrix_{MI}$/ratio), where $weighted\_matrix_{MI}$ represents the word correlation matrix before standardization, and similarly, $weighted\_matrix\_normalized_{MI}$ represents the standardized correlation matrix.

Parcellation preprocessing

A functional parcellation designed to optimize both local gradient and global similarity measures of the fMRI signal. The 400 multi-resolution areas on cerebral cortex proposed by Schaefer was used to construct the parcellation[2]. The parcellation is based on the FSL MNI152 standard space, and describes the anatomical information and associated Yeo-Krienen 17 networks for each region[3]. MATLAB code is available (https://github.com/ThomasYeoLab/CBIG/tree/master/stable_projects/brain_parcellation/Schaefer2018_LocalGlobal).

Results:

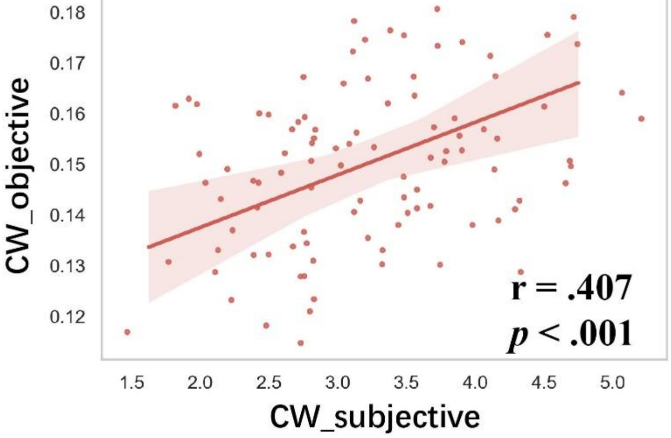

Fig. S1. Spearman correlation between subjective and objective ratings of creative writing. CW subjective and objective ratings of creative writing.

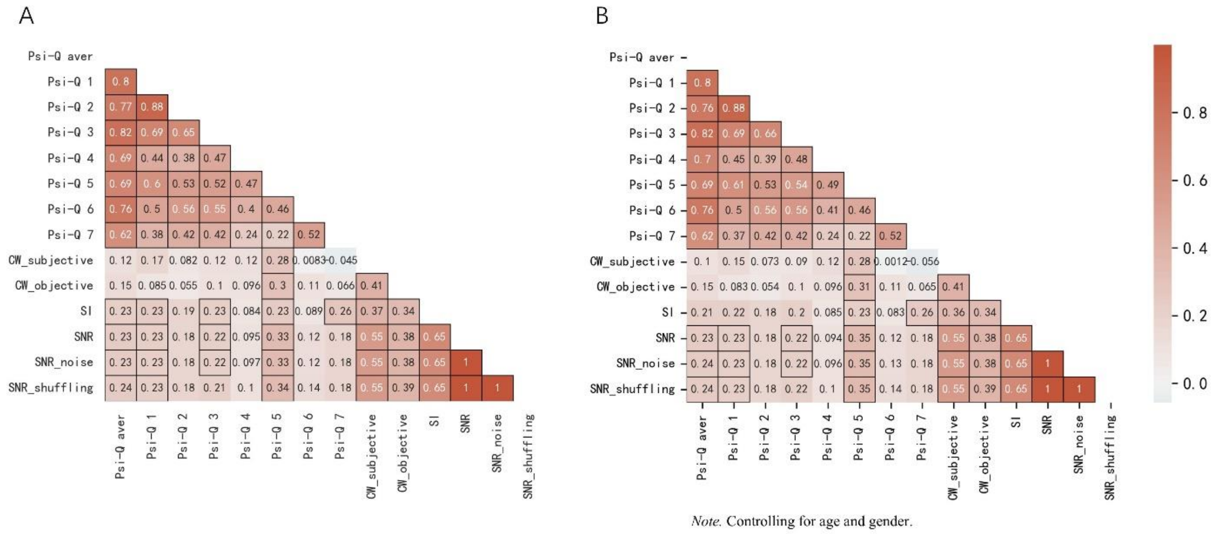

Fig. S2. Spearman's correlation heatmaps. (A) Spearman's correlations among the mental imagery vividness, creative writing scores, and semantic features. **(B)** Partial correlations controlling for age and gender. The colors represent the magnitude of the r values, with black solid outlines indicating results that are significant after FDR (False Discovery Rate) correction with $p < .05$, and numbers denote the r values. The variables are as follows: average score of Psi-Q scale, scores of the seven dimensions of Psi-Q: vision, sound, smell, taste, touch, bodily sensation, and emotional feeling, subjective and objective ratings of creative writing performance, semantic integration (SI), semantic network robustness (SNR), semantic network robustness after adding noise (SNR_noise) and shuffling links (SNR_shuffling).

Table S1. Mediation analyses.

Model	Mediator	Outcome	Indirect (ab)	95% CI	Direct (c')	Total (c)
1	SI	CW_subjective	0.1150*	[0.0263,0.2611]	0.2960*	0.4110**
2	SNR	CW_subjective	0.2343**	[0.0929,0.4184]	0.1767	0.4110**
3	SI	CW_objective	0.0025	[0.0008,0.0051]	0.0071*	0.095**
4	SNR	CW_objective	0.0034*	[0.0011,0.0067]	0.0062	0.095**

Note. All models control for age, gender. Bootstrap samples = 1000. *$p < .05$, **$p < .01$, ***$p < .001$.

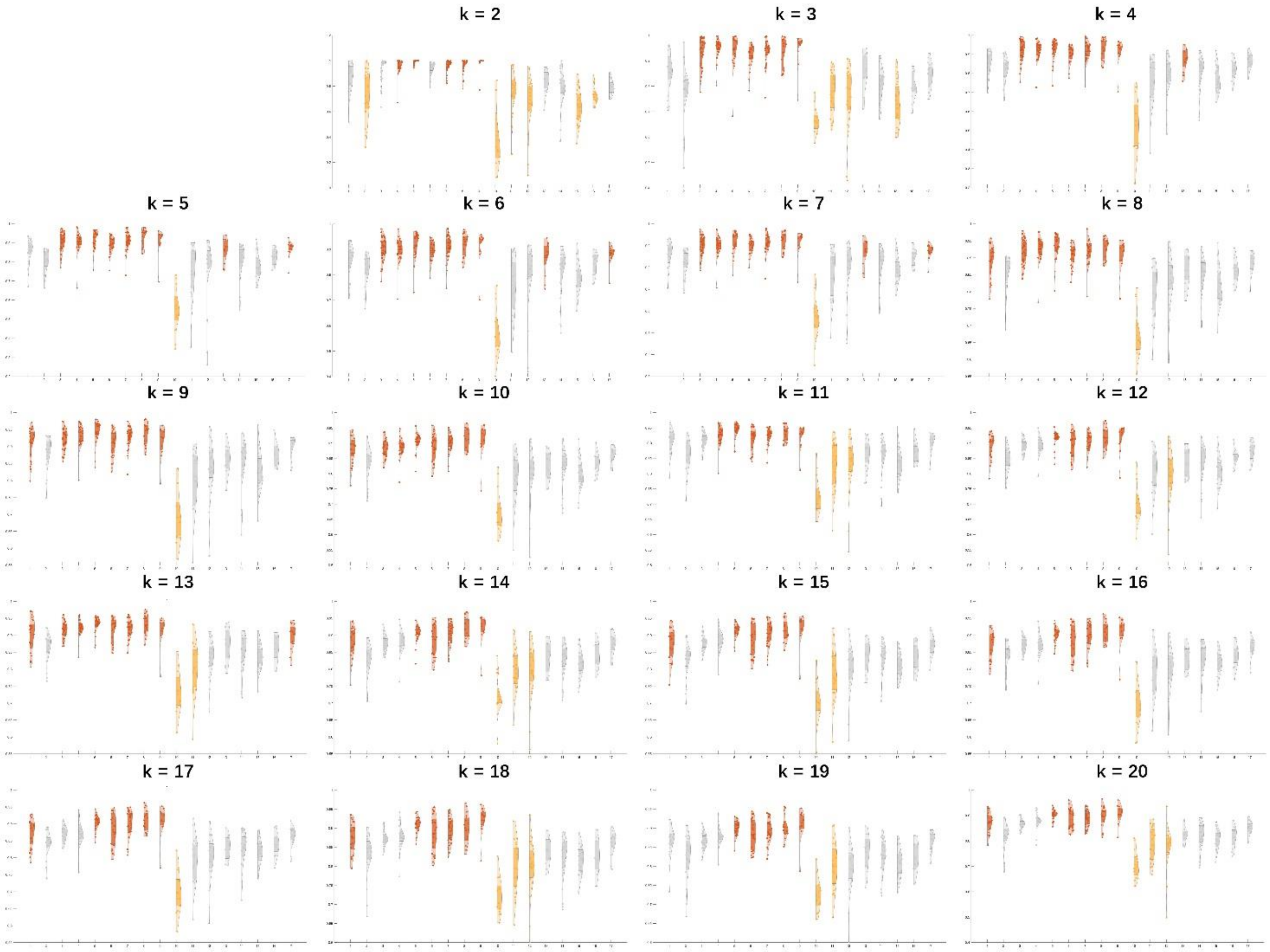

Fig. S3. Violin plots depict the distribution of edge community overlap values across all nodes within the Yeo-Krienen 17-network parcellation under mental imagery condition, analyzed at multiple scales (*k* = 2 to 20). Numbers 1-17 represent the following brain networks: 1-VIS_A, 2-VIS_B, 3-SMN_A, 4-SMN_B, 5-DAN_A, 6-DAN_B, 7-SAL_A, 8-SAL_B, 9-LIM_A, 10-LIM_B, 11-$FPCN_A$, 12-$FPCN_B$, 13-$FPCN_C$, 14-DMN_A, 15-DMN_B, 16-DMN_C, 17-TP. VIS visual network, SMN sensorimotor network, DAN dorsal attention network, SAL salience network, LIM limbic network, FPCN frontoparietal control network, DMN default mode network, TP temporal-parietal network.

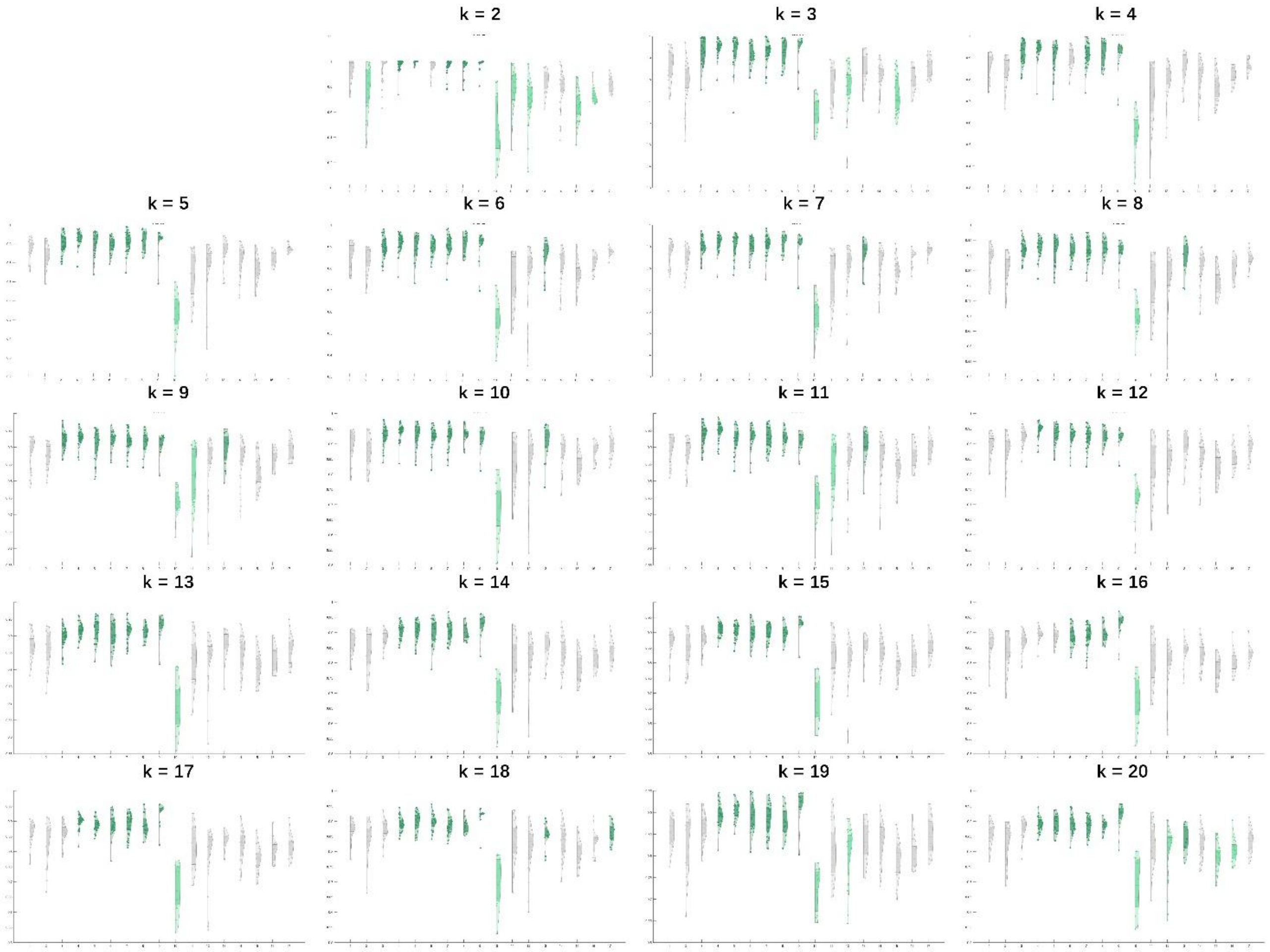

Fig. S4. Violin plots depict the distribution of edge community overlap values across all nodes within the Yeo-Krienen 17-network parcellation under semantic understanding condition, analyzed at multiple scales (*k* = 2 to 20).

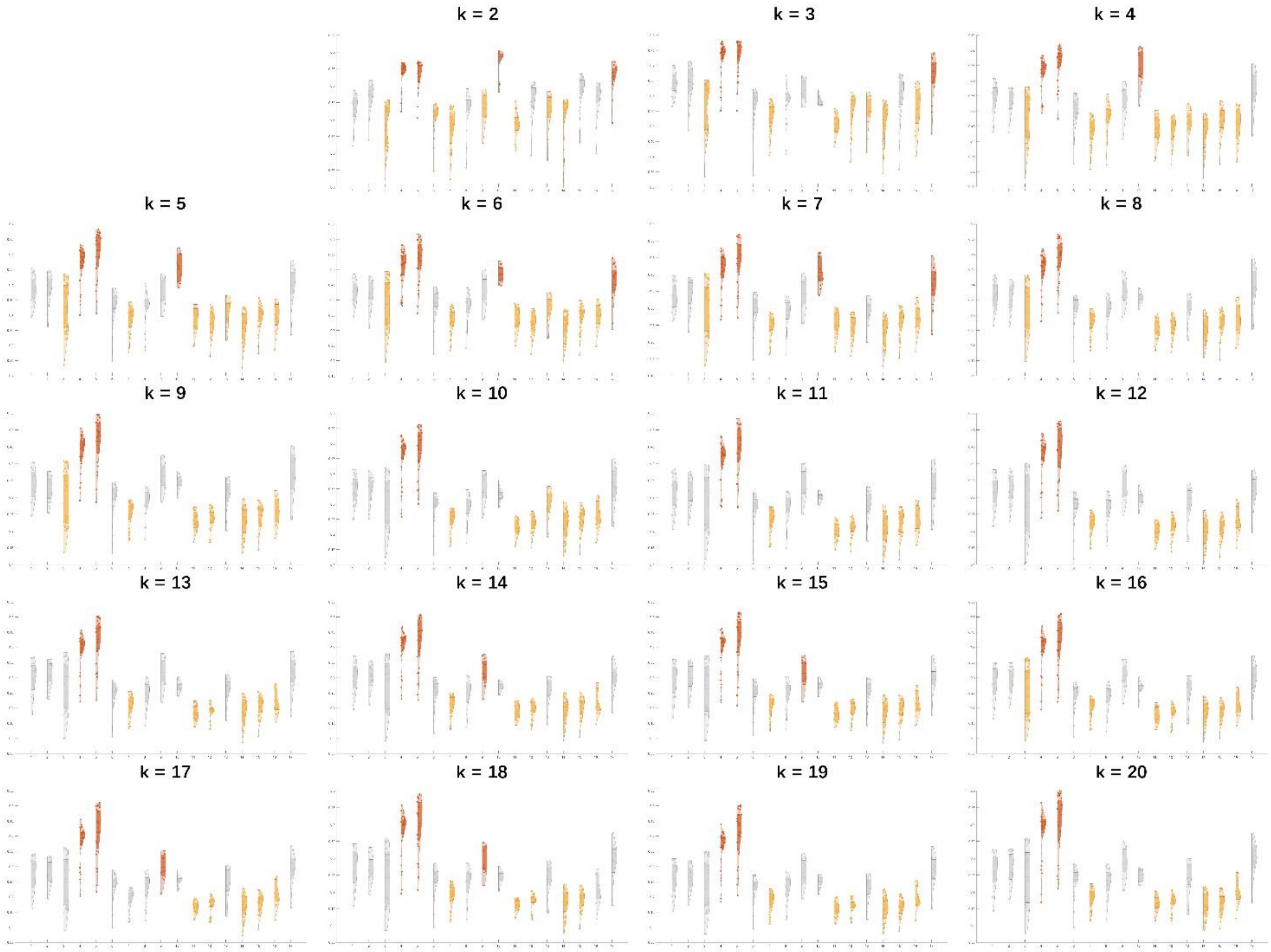

Fig. S5. Violin plots depict the distribution of edge community similarity values across all nodes within the Yeo-Krienen 17-network parcellation under mental imagery condition, analyzed at multiple scales (*k* = 2 to 20).

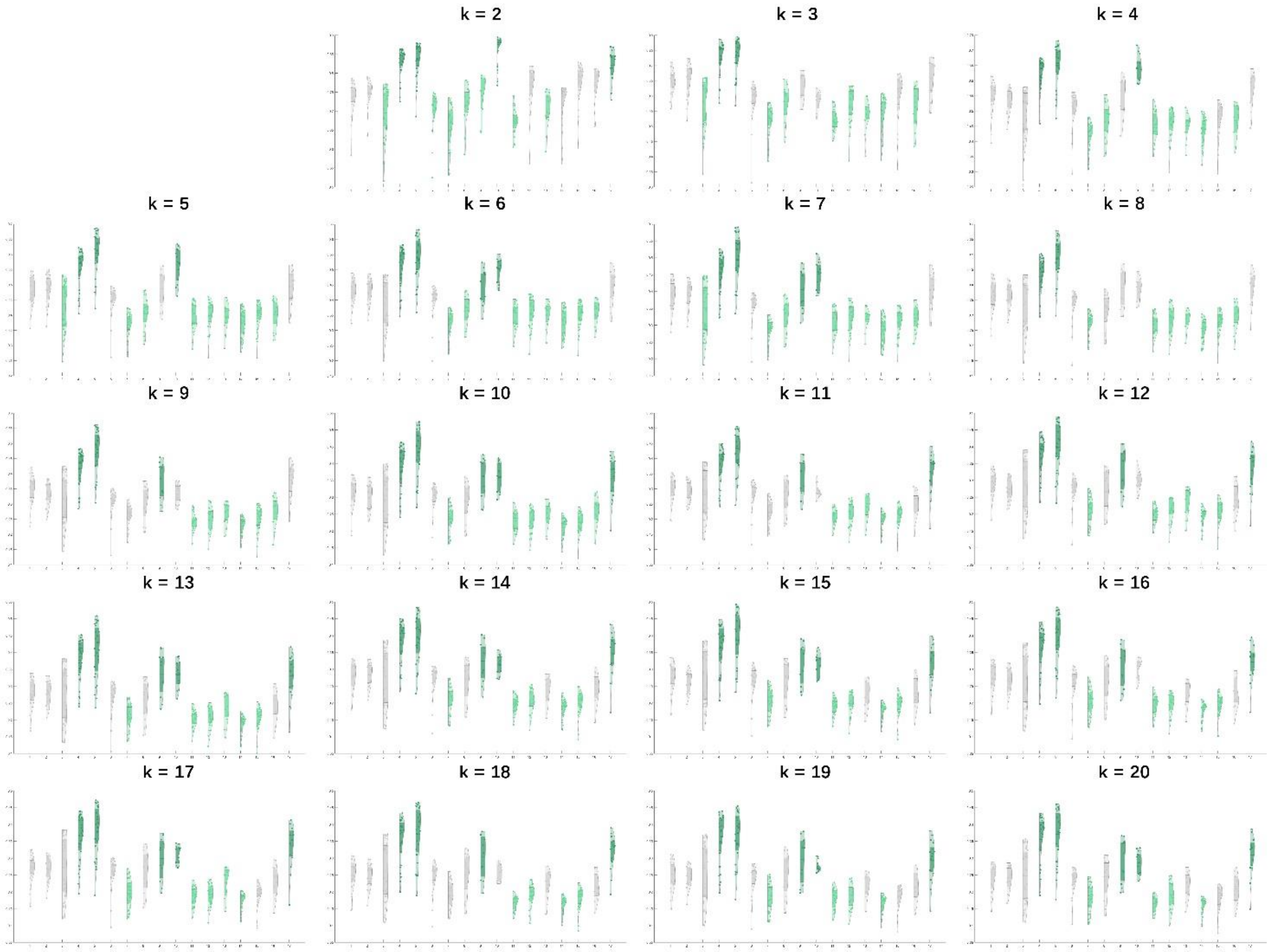

Fig. S6. Violin plots depict the distribution of edge community similarity values across all nodes within the Yeo-Krienen 17-network parcellation under semantic understanding condition, analyzed at multiple scales (*k* = 2 to 20).

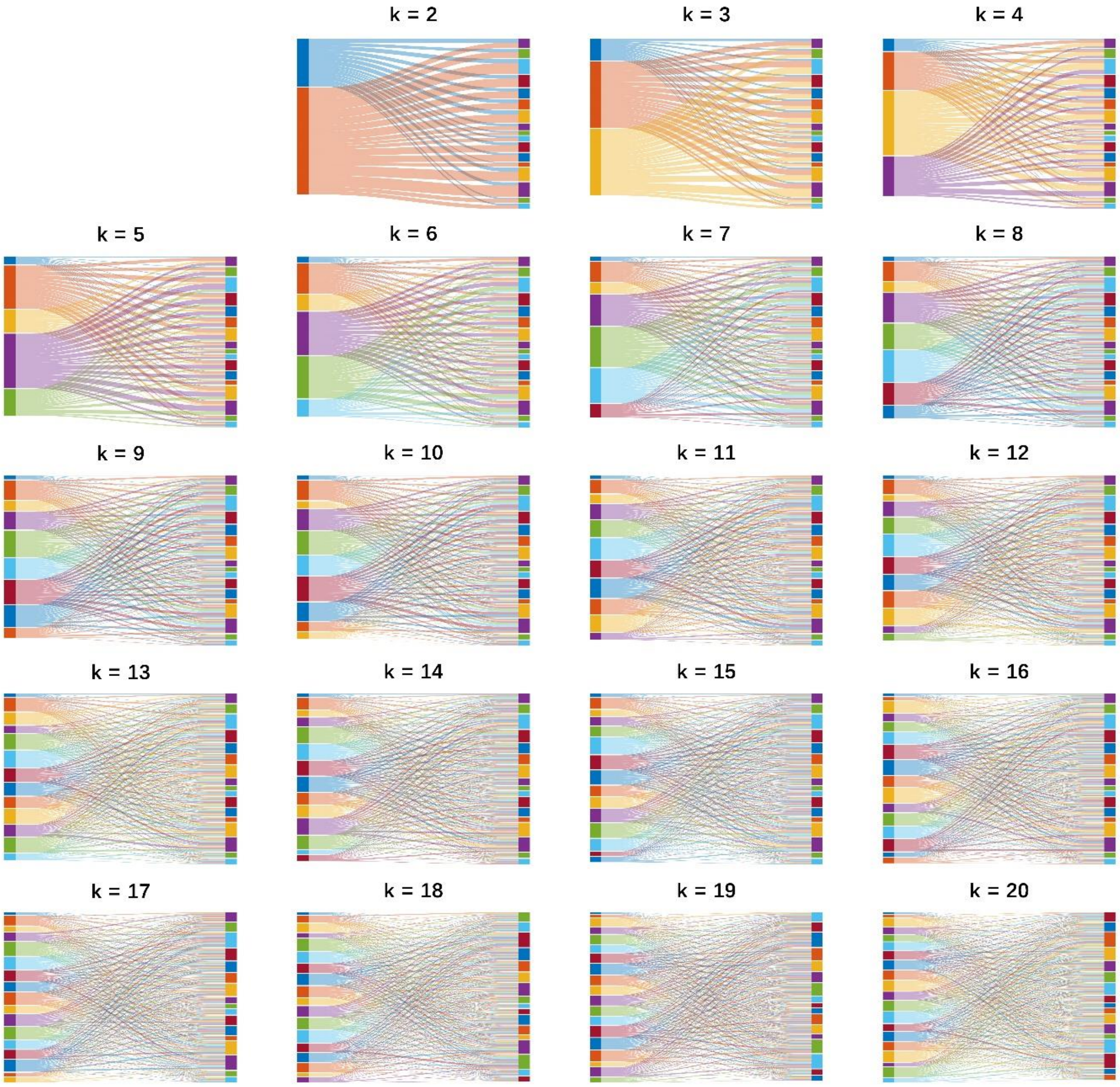

Fig. S7. Sankey diagrams showing edge-to-node community relationships, with edge communities (left) linked to their nodes' Yeo-Krienen 17-network assignments (right) across multiple scales (*k* = 2 to 20) under mental imagery condition.

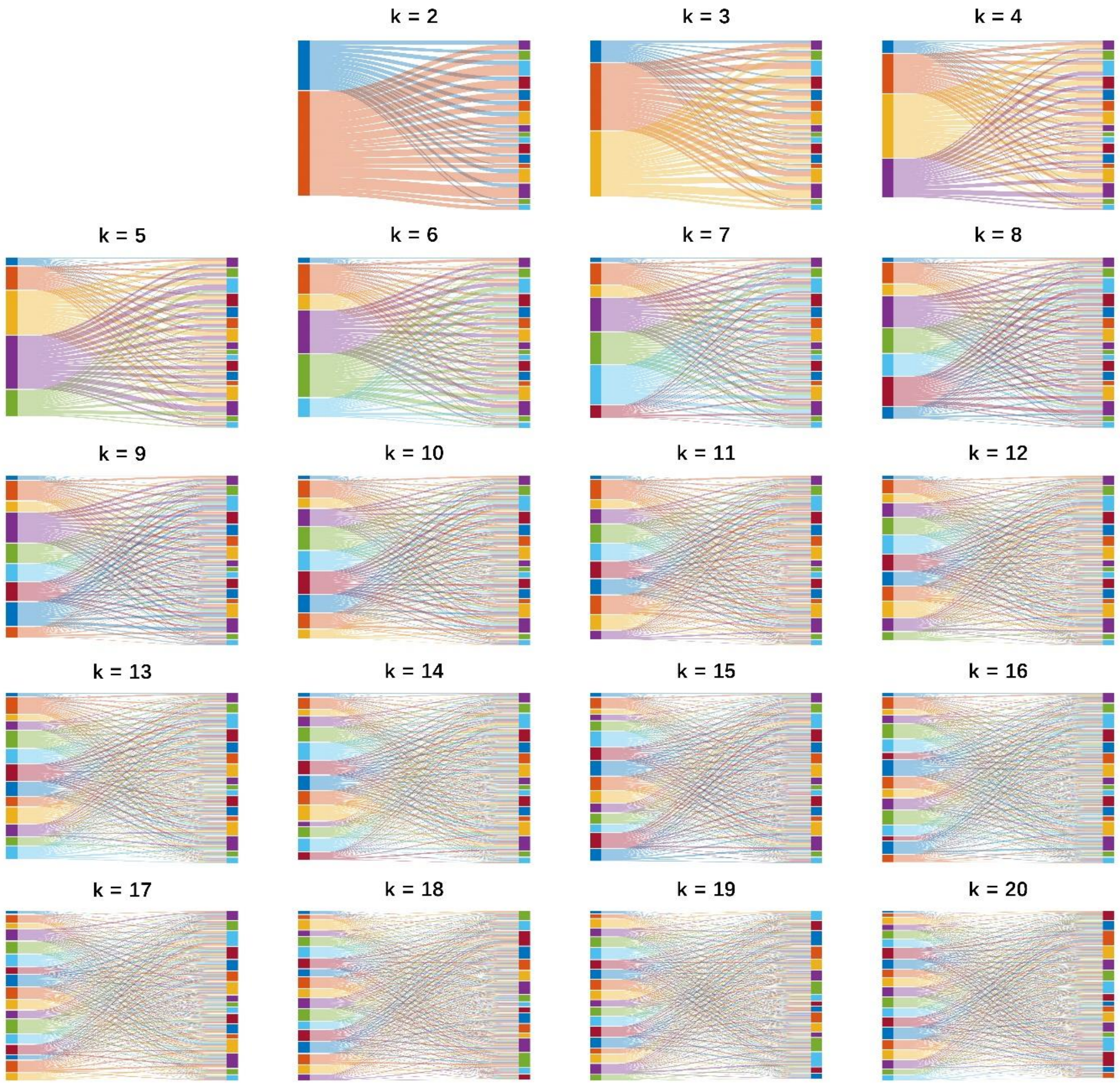

Fig. S8. Sankey diagrams showing edge-to-node community relationships, with edge communities (left) linked to their nodes' Yeo-Krienen 17-network assignments (right) across multiple scales (*k* = 2 to 20) under semantic understanding condition.

References

1. Kenett, Y. N. *et al.* Flexibility of thought in high creative individuals represented by percolation analysis. *Proc. Natl. Acad. Sci. U.S.A.* **115**, 867–872 (2018).
2. Schaefer, A. *et al.* Local-global parcellation of the human cerebral cortex from intrinsic functional connectivity MRI. *Cerebral cortex* **28**, 3095–3114 (2018).
3. Yeo, B. T. *et al.* The organization of the human cerebral cortex estimated by intrinsic functional connectivity. *Journal of neurophysiology* **106**, 1125–1165 (2011).
