# Supplementary material for: Cognitive and neural mechanisms of mental imagery supporting creative cognition
Source: Commun Biol. 2025 Sep 30;8:1386. doi: 10.1038/s42003-025-08513-x (PMC12484779; doi:10.1038/s42003-025-08513-x)
Supplement: Supplementary file 3 — Description of Additional Supplementary Files [file 42003_2025_8513_MOESM3_ESM.pdf]

Description of Additional Supplementary Files

File name: Supplementary Data 1

Description: Results of the Dwass-Steel-Critchlow-Fligner test

File name: Supplementary Data 2

Description: The source data behind the figures in the paper
