# Supplementary material for: Cognitive and neural mechanisms of mental imagery supporting creative cognition
Source: Commun Biol. 2025 Sep 30;8:1386. doi: 10.1038/s42003-025-08513-x (PMC12484779; doi:10.1038/s42003-025-08513-x)
Supplement: Supplementary file 6 — Reporting summary [file 42003_2025_8513_MOESM6_ESM.pdf]

nature portfolio

Corresponding author(s): Jiang Qiu, Yoed N. Kenett , and Jiangzhou Sun

Last updated by author(s): Jun 23, 2025

Reporting Summary

Nature Portfolio wishes to improve the reproducibility of the work that we publish. This form provides structure for consistency and transparency in reporting. For further information on Nature Portfolio policies, see our Editorial Policies and the Editorial Policy Checklist.

Statistics

For all statistical analyses, confirm that the following items are present in the figure legend, table legend, main text, or Methods section.

n/a	Confirmed	
☐	☒	The exact sample size (*n*) for each experimental group/condition, given as a discrete number and unit of measurement
☐	☒	A statement on whether measurements were taken from distinct samples or whether the same sample was measured repeatedly
☐	☒	The statistical test(s) used AND whether they are one- or two-sided<br>*Only common tests should be described solely by name; describe more complex techniques in the Methods section.*
☐	☒	A description of all covariates tested
☐	☒	A description of any assumptions or corrections, such as tests of normality and adjustment for multiple comparisons
☐	☒	A full description of the statistical parameters including central tendency (e.g. means) or other basic estimates (e.g. regression coefficient) AND variation (e.g. standard deviation) or associated estimates of uncertainty (e.g. confidence intervals)
☐	☒	For null hypothesis testing, the test statistic (e.g. *F*, *t*, *r*) with confidence intervals, effect sizes, degrees of freedom and *P* value noted<br>*Give P values as exact values whenever suitable.*
☒	☐	For Bayesian analysis, information on the choice of priors and Markov chain Monte Carlo settings
☒	☐	For hierarchical and complex designs, identification of the appropriate level for tests and full reporting of outcomes
☐	☒	Estimates of effect sizes (e.g. Cohen's *d*, Pearson's *r*), indicating how they were calculated

Our web collection on statistics for biologists contains articles on many of the points above.

Software and code

Policy information about availability of computer code

Data collection	Behavioral and neuroimaging data were obtained at Southwest University. The behavior data was obtained by a professional questionnaire tool (https://www.wjx.cn/). The neuroimaging data was obtained by a 3T Siemens Prisma scanner (Erlangen, Germany).
Data analysis	The Spearman's correlation analysis, mediation analyses and Kruskal-Wallis test were analyzed by jamovi 2.3.28. The one-sample and paired-samples t-test analyses were analyzed by IBM SPSS Statistics 26. The objective scoring, semantic integration and semantic network robustness were analyzed by Python 3.10. Python code for semantic integration is publicly available (https://osf.io/ath2s/). The MRI data were preprocessed using dpabi (version 8.1) in MATLAB (2021a). MATLAB code for edge graph construction and community analysis is publicly available (https://github.com/brain-networks/edge-centric_demo). Custom Python and MATLAB scripts are available upon request to the corresponding author (J.Q.).

For manuscripts utilizing custom algorithms or software that are central to the research but not yet described in published literature, software must be made available to editors and reviewers. We strongly encourage code deposition in a community repository (e.g. GitHub). See the Nature Portfolio guidelines for submitting code & software for further information.

Data

Policy information about availability of data

All manuscripts must include a data availability statement. This statement should provide the following information, where applicable:

- Accession codes, unique identifiers, or web links for publicly available datasets
- A description of any restrictions on data availability
- For clinical datasets or third party data, please ensure that the statement adheres to our policy

All data are available upon request to the corresponding author (J.Q.).

Research involving human participants, their data, or biological material

Policy information about studies with human participants or human data. See also policy information about sex, gender (identity/presentation), and sexual orientation and race, ethnicity and racism.

Reporting on sex and gender	All participants' gender was based on self-reported sex in this study. We did not specially considersex in the study design and did not analyze it.
Reporting on race, ethnicity, or other socially relevant groupings	All participants in this study were from China.
Population characteristics	Study 1: 98 (78 females; mean age 21.63 years ± 2.16).<br>Study 2: 68 (52 females; mean age = 21.12 years ± 1.66).<br>Study 3:29 (21 females; mean age 21.48 years ± 2.33).
Recruitment	All participants were recruited via online advertisements.
Ethics oversight	All procedures were approved by the institutional review board of Southwest University, and participants were healthy with no history of neurological or psychiatric conditions, no metal implants, and no claustrophobia. Informed consent was obtained, and participants were compensated upon completion.

Note that full information on the approval of the study protocol must also be provided in the manuscript.

Field-specific reporting

Please select the one below that is the best fit for your research. If you are not sure, read the appropriate sections before making your selection.

☐ Life sciences ☒ Behavioural & social sciences ☐ Ecological, evolutionary & environmental sciences

For a reference copy of the document with all sections, see nature.com/documents/nr-reporting-summary-flat.pdf

Behavioural & social sciences study design

All studies must disclose on these points even when the disclosure is negative.

Study description	A qualitative cross-sectional study.
Research sample	Southwest University undergraduates.<br>Study 1: 98 (78 females; mean age 21.63 years ± 2.16).<br>Study 2: 68 (52 females; mean age = 21.12 years ± 1.66).<br>Study 3:29 (21 females; mean age 21.48 years ± 2.33)
Sampling strategy	Study 1 was based on convenience sampling. Study 2 was calculated in G*Power to detect large effects (d = 0.5) at α = .05 (two-tailed) for paired t-tests. Study 3 (fMRI) was budget-driven.
Data collection	Data of Studies 1 and 2 were collected on computers provided by the Faculty of Psychology of Southwest University. Data of Study 3 was obtained by a 3T Siemens Prisma scanner (Erlangen, Germany). Researchers were blind to experimental condition during data collection.
Timing	Study 1: July to September 2023.<br>Study 2: November 2023.<br>Study 3: January 2024.
Data exclusions	Study 1: nine participants were excluded for errors on the screening questions, one for an excessively brief response time (576 seconds), and one for an outlier Psi-Q touch score, which fell outside the 25th - 75th percentile distribution, with the score being 2.

	Study 2: no participants were excluded.<br>Study 3: one was excluded due to scanning issues.
Non-participation	None.
Randomization	Participants were randomly allocated into experimental groups.

Reporting for specific materials, systems and methods

We require information from authors about some types of materials, experimental systems and methods used in many studies. Here, indicate whether each material, system or method listed is relevant to your study. If you are not sure if a list item applies to your research, read the appropriate section before selecting a response.

Materials & experimental systems

n/a	Involved in the study
☒	☐ Antibodies
☒	☐ Eukaryotic cell lines
☒	☐ Palaeontology and archaeology
☒	☐ Animals and other organisms
☒	☐ Clinical data
☒	☐ Dual use research of concern
☒	☐ Plants

Methods

n/a	Involved in the study
☒	☐ ChIP-seq
☒	☐ Flow cytometry
☐	☒ MRI-based neuroimaging

Plants

Seed stocks	n/a
Novel plant genotypes	n/a
Authentication	n/a

Magnetic resonance imaging

Experimental design

Design type	Block design.
Design specifications	20 blocks. The length of each block: 87 seconds. The length of each interval: 12 seconds.
Behavioral performance measures	Oral reports have been recorded to ensure participants have accomplish the task.

Acquisition

Imaging type(s)	Functional and structural.
Field strength	3 Tesla
Sequence & imaging parameters	Study 3 utilized magnetization prepared rapid acquisition gradient echo (MPRAGE) to acquire structural images, with scan parameters including: repetition time (TR) = 2530 ms, echo time (TE) = 2.98 ms, flip angle (FA) = 7°, field of view (FOV) = 256×224 mm2, voxel size = 0.5x0.5x1.0 mm3, slice thickness = 1.0 mm. Task-based fMRI data were collected using a gradient-echo planar imaging (EPI) sequence, with scan parameters of TR= 1000 ms, TE= 30 ms, FA = 73°, FOV = 195×195 mm2, voxel size = 2.5×2.5×2.5 mm3, slice thickness = 2.5 mm.
Area of acquisition	Whole-Brain

Diffusion MRI ☐ Used ☒ Not used

Preprocessing

Preprocessing software	dpabi (version 8.1)
Normalization	Linear realignment to MNI152Nlin6Asym spaces.
Normalization template	MNI standard space.
Noise and artifact removal	Regression covariates included five noise principal components from white matter (WM) and cerebrospinal fluid (CSF), Friston's 24 head motion parameters, and the first-order linear effect. Bandpass filtering (0.01-0.1Hz) was applied to reduce the effects of low-frequency drift and high-frequency physiological noise.
Volume censoring	No volume was discarded.

Statistical modeling & inference

Model type and settings	n/a
Effect(s) tested	n/a

Specify type of analysis: ☒ Whole brain ☐ ROI-based ☐ Both

Statistic type for inference (See Eklund et al. 2016)	n/a
Correction	n/a

Models & analysis

n/a	Involved in the study
☐	☒ Functional and/or effective connectivity
☐	☒ Graph analysis
☒	☐ Multivariate modeling or predictive analysis

Functional and/or effective connectivity	Correlation.
Graph analysis	Weighted graph; edge community overlap and edge community similarity.
